# Ice Nucleation Properties of Ice-binding Proteins from Snow Fleas

**DOI:** 10.3390/biom9100532

**Published:** 2019-09-25

**Authors:** Akalabya Bissoyi, Naama Reicher, Michael Chasnitsky, Sivan Arad, Thomas Koop, Yinon Rudich, Ido Braslavsky

**Affiliations:** 1 The Robert H. Smith Faculty of Agriculture, Food and Environment, Institute of Biochemistry, Food Science, and Nutrition, The Hebrew University of Jerusalem, Rehovot 7610001, Israel; akalabya.bissoyi@mail.huji.ac.il (A.B.); Michael.Chasnitsky@mail.huji.ac.il (M.C.); sivan.benbassat@mail.huji.ac.il (S.A.); 2 Department of Earth and Planetary Sciences, The Weizmann Institute of Science, Rehovot 7610001, Israel; reicher.naama@icloud.com (N.R.); yinon.rudich@weizmann.ac.il (Y.R.); 3 Bielefeld University, Faculty of Chemistry, D-33615 Bielefeld, Germany; thomas.koop@uni-bielefeld.de

**Keywords:** ice nucleation, antifreeze proteins, ice binding proteins, collembola in Israel, *Hypogastruridae*, thermal hysteresis, hyperactive

## Abstract

Ice-binding proteins (IBPs) are found in many organisms, such as fish and hexapods, plants, and bacteria that need to cope with low temperatures. Ice nucleation and thermal hysteresis are two attributes of IBPs. While ice nucleation is promoted by large proteins, known as ice nucleating proteins, the smaller IBPs, referred to as antifreeze proteins (AFPs), inhibit the growth of ice crystals by up to several degrees below the melting point, resulting in a thermal hysteresis (TH) gap between melting and ice growth. Recently, we showed that the nucleation capacity of two types of IBPs corresponds to their size, in agreement with classical nucleation theory. Here, we expand this finding to additional IBPs that we isolated from snow fleas (the arthropod Collembola), collected in northern Israel. Chemical analyses using circular dichroism and Fourier-transform infrared spectroscopy data suggest that these IBPs have a similar structure to a previously reported snow flea antifreeze protein. Further experiments reveal that the ice-shell purified proteins have hyperactive antifreeze properties, as determined by nanoliter osmometry, and also exhibit low ice-nucleation activity in accordance with their size.

## 1. Introduction

Collembola (also referred to as springtails) are the world’s most abundant hexapods (arthropods and insects) and can be found in diverse habitats, from the tropics to the poles [1]. Collembola, an ancient group of organisms, forms a separate class within the phylum Arthropoda. These arthropods can survive at low temperatures, and the physiological processes behind this survival are well documented. Collembola species that are found in snowy environments are also commonly called snow fleas due to their jumping habit and dark color, although these organisms are not fleas.

Studies on Collembola from Israel have been conducted for almost 100 years [2], mostly in Mount Carmel over the past two decades. Currently, 128 species of Collembola from Israel are known. A survey of the geographical regions of Israel showed that the highest number of species and individuals are located in the Mount Hermon area, where temperatures reach below −10 °C [3]. A new springtail, based on phylogenetic analysis, which we recently found in the snow in the same area, is studied in this report. These springtails are cold-tolerant organisms. In contrast to other arthropods, springtails cannot prevent low-temperature injury by hibernation. Instead, they undergo physiological changes that reduce their freezing temperature and enable their survival at low temperatures [4]. Furthermore, numerous Collembola species use protective desiccation as a strategy to avoid freezing in cells. Trehalose has an important contribution to this strategy as a cryoprotectant that maintains membrane integrity [5]. Springtails can also decrease the freezing temperatures by producing antifreeze proteins (AFPs) [6,7]. These AFPs, which are small ice-binding proteins (IBPS), lower the freezing point of a solution by up to a few degrees due to their ice-binding property that causes a difference between the non-equilibrium freezing temperature and the melting temperatures. This is called the thermal hysteresis (TH) gap [8], see Section 3.4 below. Another way to mitigate freezing injury is by promoting extracellular ice nucleation at low supercooling, which reduces the rate of ice formation inside cells [9]. Such ice-nucleating ability has been observed in various studies of the Arctic springtail *Onychiurus arcticus* [9,10] and the Antarctic midge *Belgica antarctica* [11,12].

In the current study, we identified the species of the snow flea we found in the Hermon mountain, compared their protein characteristics to known snow flea proteins [6,7], and characterized their relevant ice activity properties. In particular, we studied the ice nucleation ability of their AFPs. This property was interesting due to the recent study by Eickhoff et al. [13], which showed that type III AFPs from fish and AFPs from the Tenebrio molitor beetle (TmAFP) promote ice nucleation in addition to their antifreeze activity. 

## 2. Methods

### 2.1. Sample Collection and Preparation

Snow fleas were collected from a valley on Mount Hermon, Israel (33.291 N, 35.755 E) at 1450 m above sea level in January 2019 using insect aspirators. Approximately 2000 specimens of Collembola (a mixture of adults and juveniles) were kept in a few glass jars (one jar is shown in Figure S1) and transported on dry ice. The samples, kept at −80 °C, were subsequently lyophilized and stored at 4 °C in sealed vials. The total mass of the samples after lyophilizing was approximately 120 mg. While having observed Collembola multiple times over many years (2015–2019) at this collection location and at a nearby location (33.308 N, 35.772 E), Collembola has not been observed during several attempts at altitudes (33.307 N, 35.784 E) higher than 2000 m above sea level. See Figure S1 and Movie S1 for images and movies of the snow fleas at the collection location. 

### 2.2. Species Identification

A blood and tissue kit (DNeasyBlood & Tissue Kit; Qiagen, Germantown, MD, USA) was used to extract genomic DNA from mitochondria-rich tissues. The 710 bp region of the mitochondrial genome (cytochrome oxidase I (COI)) barcoding region was amplified using a polymerase chain reaction (PCR). The primer sequences used were:

“LOC1490”5′-ATTCAACCAATCATAAAGATATTGG-3′ and 

“HCO2198”5′-TAAACTTCTGGATGTCCAAAAAAC-3′ [14]. 

Briefly, the reactions were carried out in 10 μL volumes in a polymerase chain reaction (PCR) thermal cycler (5333 MasterCycler; Eppendorf, Hamburg, Germany) equipped with a 96-well under the following conditions: A first cycle of 95 °C for 5 min followed by 5 cycles of 94 °C for 30 s, annealing at 45 °C for 30 s and 72 °C for 1 min, then 39 cycles of 94 °C for 30 s, annealing at 60 °C for 30 s, and, finally, 72 °C for 5 min. The PCR products were viewed with 1.2% agarose (ChemiDoc MP; in Bio-Rad, Hercules, CA, USA), Figure S2, and the sequencing was then conducted by HyLabs, Rehovot, Israel, as presented in the Supplementary Data. Online software from the National Center for Biotechnology Information’s (NCBI) basic local alignment search tool (BLAST) was used to check the PCR products. The COI sequences from the samples were compared with reference sequences from the NCBI’s and the Barcode of Life Data System’s (BOLD) databases to identify the collected specimens [15,16]. Phylogenetic trees were constructed from all the COI sequences using the Kimura 2-parameter model, NJ K2P, from the software Figtree, Version 1.4.4, [17] (Andrew Rambaut, Edinburgh, UK). 

### 2.3. Morphological Analysis Using Cryogenic Scanning Electron Microscopy, Cryo-SEM

The morphological analysis of the springtail samples were carried out using a Cryo-SEM device (7800 FE-SEM; JEOL, Tokyo, Japan) at a high voltage of 5 kV. All the samples were rapidly frozen to reduce ice-crystal damage and improve the preservation of the specimens. The specimens were subsequently sputter-coated with carbon to avoid any charging effects. 

### 2.4. Centrifugal Filtration

**Figure 1:**
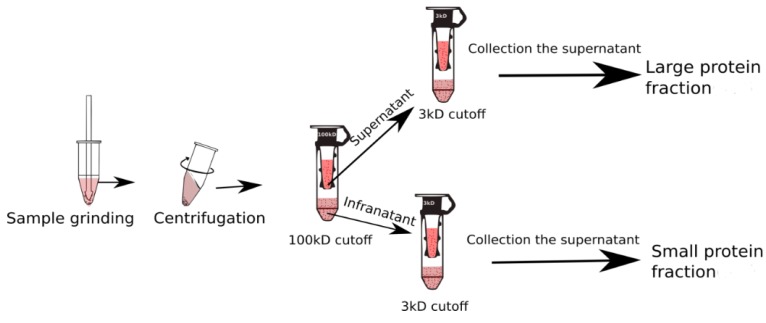
Schematic representation of the separation process used in the current study using the membrane cut-off base principle.

The snow fleas were homogenized in 50 mM Tris-HCl, 150 mM NaCl pH 7, and 1 mM phenylthiocarbamide, containing phenylmethylsulfonyl fluoride (PSMF), and subsequently centrifuged to separate the protein fraction from the extracellular matrices. After centrifugation, the supernatant was added to 50 mM Tris-HCl pH 8 buffer. A 100 kDa molecular weight cut-off centrifugal filter (Vivaspin 500, MWCO 100; Sartorius, Goettingen, Germany) was used to separate molecules of distinct sizes in the crude snow flea extract (Figure 1). A stock solution was initially centrifuged at 5000 rpm for ~60 min, and the supernatant was occasionally shaken every 5 min, approximately. The filtrates were subsequently separated from the tube and stored individually [18]. 

### 2.5. Ice-Affinity Purification

**Figure 2:**
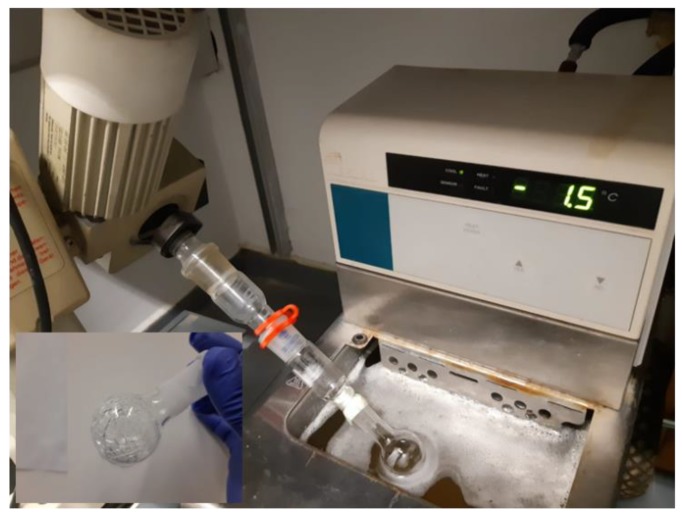
Ice-shell purification using a 10 mL round bottom flask.

The snow flea AFP was purified using the ice-shell affinity purification method [19]. We used a small (10 mL) round bottom flask for the purification, as shown in Figure 2. Initially, a 1 mL ice shell of double distilled water (DDW) was formed in the flask using a cold bath. A total of 40 mg of <100 kDa crude lysate solution was diluted in 50 mM of Tris-HCl and 150 mM of NaCl pH 7 buffer to a final volume of 1 mL. This solution was added to the flask, and half of it was frozen. The unfrozen liquid fraction (LF) was removed from the flask, and the 1.5 mL ice fraction (IF1) was melted. A total of 1 mL of the IF1 was then added to a new 1 ml DDW ice shell, and the process was repeated (IF2) in order to fully purify the snow flea AFP from the solution. After melting the 1.5 mL IF2, the solution was concentrated using a centrifugal concentrator (Vivaspin 20; Sartorius, Blenheim, UK).

### 2.6. Sodium Dodecyl Sulfate-Polyacrylamide Gel Electrophoresis (SDS-PAGE), Mass Spectrometry, and Protein Concentration Determination

Sodium dodecyl sulfate-polyacrylamide gel electrophoresis (SDS-PAGE) was conducted in a 6% stacking gel and a 16% solving gel following a previously-used procedure [20]. Mass spectrometry analysis was performed on a Microflex LT mass spectrometer (Bruker Daltonics, Billerica, MA, USA) using FlexControl software. The protein concentration was determined against bovine serum albumin (BSA) as a standard using a bicinchoninic acid assay (BCA) protein estimation kit (Thermo Scientific, Waltham, MA, USA). Absorption was measured at 550 nm using a microplate reader (Infinite F200; Tecan, Switzerland). For a blank, we used 50 mM of Tris-HCl and 150 mM of NaCl pH 7 buffer [20].

### 2.7. Infrared Spectroscopy

Fourier-transform infrared (FTIR) spectroscopy of the samples was undertaken using an FTIR spectrometer (Nicolet 6700; Thermo Scientific) in total reflection mode. The experiment was performed as previously discussed in the literature [21,22]. Spectra were recorded from 800 cm^−1^ to 1600 cm^−1^ with a spectral resolution of 1 cm. All the samples were air-dried for 30 min inside a laminar flow bench before the FTIR analysis.

### 2.8. Secondary Structure Estimation

The >100 kDa fraction and the <100 kDa that was further purified on ice-shell fraction (that contained the ice-binding proteins (IBPs)) were dialyzed against 20 mM sodium phosphate at pH 7. Circular dichroism (CD) spectroscopy (Chirascan V100; Applied Photophysics Ltd., Leatherhead, UK) was performed at 4 °C. The spectrum was analyzed using DichroWeb [23,24].

### 2.9. Thermal Hysteresis Activity Measurement

We used a custom-designed nanoliter osmometer system with a temperature controller (3040; Newport Corp., Irvine, CA, USA) and a customized cooling stage, previously described by [8]. The temperature of the cooling stage was regulated by a LabVIEW program developed in our laboratory. Each droplet was measured twice to ensure consistent results. 

### 2.10. Ice Nucleation Measurement

Ice nucleation measurements were performed using the Weizmann Supercooled Droplets Observation on a Microarray (WISDOM) setup, detailed by [13,25,26]. A total of 90 μm diameter droplets containing a solution of AFPs were generated and trapped in a microfluidics device, placed onto a cold stage (THMS600; Linkam), and coupled to an optical microscope (BX-51; Olympus) with ×10 magnification in transmission mode for the freezing experiments. The droplets were surrounded by a 2 wt% mixture of emulsifier (Span 80; Sigma-Aldrich, Merck KGaA, Darmstadt, Germany) in light mineral oil (Sigma-Aldrich), which kept the droplets stable during the freezing experiments and prevented interference with the neighboring droplets. The droplets were cooled at a rate of 1 °C min^−1^ and the freezing temperature was determined automatically using an image analysis LabVIEW program for each individual droplet based on the sudden decrease in the droplet’s brightness during freezing.

### 2.11. Ice Nucleation Analysis

The experimental ice nucleation data were analyzed as described previously [13]. From the measured curves of the cumulative frozen fraction as a function of temperature, we determined the median freezing temperature, at which 50% of the droplets froze upon cooling, T_50_, for pure water droplets, T_50_(wat); pure buffer droplets, T_50_(buf), and droplets containing various concentrations of IBP in the buffer, T_50_(IBP). The Tris buffer had a significant effect on the T_50_ temperatures (T_50_(wat) = −35.7 ± 0.4 °C and T_50_(buf) = −38.2 ± 0.4 °C) because the solutes decreased both the ice melting point and the homogeneous ice nucleation temperature [25,27]. Therefore, to compare the heterogeneous ice nucleation temperatures of the IBP, T_het_(IBP) with previous measurements, as well as with theoretical predictions of the critical ice embryo size in pure water, the measured ice nucleation temperatures were corrected for the effect of the buffer, similar to previous studies [27,28] as follows:

(1) $$T_{het}\left(IBP\right)=\;T_{50}\left(IBP\right)+\;\left[T_{50}\left(wat\right)-\;T_{50}\left(buf\right)\right]$$

Hence, the T_het_(IBP) is slightly shifted, similar to the effect by which the buffer decreases the T_50_(wat) of pure water. Note that this treatment also accounts for the effect on freezing temperature, with different buffers or different buffer concentrations. It also allows for a comparison of the heterogeneous ice nucleation temperature and the corresponding critical ice embryo size determined from classical nucleation theory, which is usually calculated for pure water and not for buffer solution. Moreover, a comparison between previously measured ice nucleation temperatures of IBPs and the critical ice embryo from classical nucleation theory (CNT) requires an estimate of the three-dimensional size of the IBP, which was previously estimated from its molecular weight and the protein density, assuming a cubic protein shape, see Eickhoff et al. (2019) for details and corresponding parameterizations [13]. For the current proteins, we used the estimated size of the ice binding site of the related protein [6,29,30], see further below.

## 3. Results

### 3.1. Gene Barcoding 

The mitochondrial genome COI 676-bp fragment has been sequenced (See Methods Section 2.2); however, the 569-bp sequence was used in the current study to reduce any sequencing discrepancy (as shown in the Supplementary Data). No insertions or deletions were identified in any of the sequences. The nucleotide composition was shown on an Adenine and Thiamine **(**AT) bias (A = 138, T = 219, C = 106, and G = 106), and the Guanine and Cytosine (GC) content was 37.3%. An AT nucleotide bias frequently found in hexapods was also identified. The percentage of AT (62.7%) found in the current study was close to the percentage of 63–64% reported for Antarctic Collembola [31,32] and significantly less than the 70–75% reported for some insect taxa [31,32].

**Figure 3:**
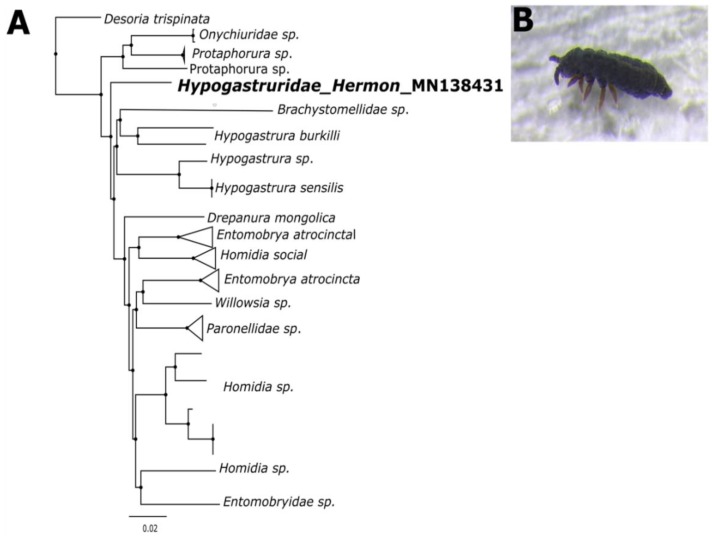
(**A**) Phylogenetic analysis using the neighbor-joining method of uncorrected p-distances based on the 596-bp fragment of the COI gene. (**B**) Image of a Collembola on snow prior to collection.

The COIs were identified at the species level in the GenBank and BOLD databases. A single neighbor-joining (NJ) tree, featuring the captured Collembola COI sequence, along with reference COI genes from the NCBI database, showed five major clusters corresponding to the Protaphorura, Brachystomellidae, Hypogastrura, Homidia, and Entomobrya families. There were no anomalies among any of these classes, and all the recognized reference samples clustered properly (Figure 3). The sequence was also searched against the BOLD database, which tentatively identified the springtail as Poduromorpha and the highest similarity with species *Hypogastruridae* GEN sp. DPCOL27818. We deposited the COI sequence to the GenBank database and it got the accession number Hypogastruridae_COI_Mt_Hermon_Israel MN138431

### 3.2. Morphological Analysis

**Figure 4:**
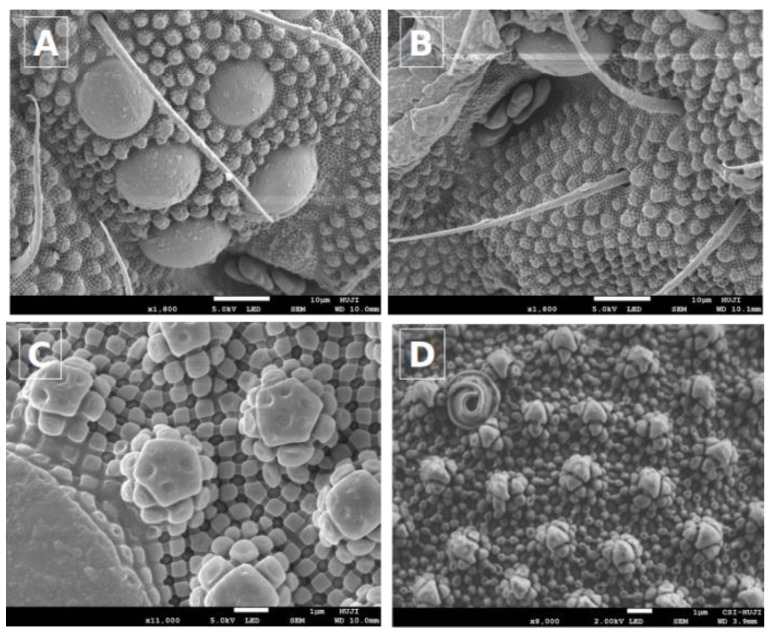
SEM images of a springtail. (**A**) and (**B**) magnified view of the compound eyes of a springtail, including microscale hairs and so-called secondary granules, with a scale bar of 10 microns. (**C**) Magnified image of a snow flea cuticle showing the network pattern of lower primary granules located between the secondary granules. (**D**) Magnified view of a springtail’s dorsal cuticle. The scale bar for (**C**) and (**D**) is 1 micron.

The specific feature of the Collembolan’s cuticle is the comb-like structures described by Helbig et. al. [33]. These structures primarily consist of main granules in the form of triangles linked by ridge-forming hexagons (Figure 4), which also include rhombic patterns and secondary granules [33]. This structure has important functions, such as water-repellence, antimicrobial effects, and self-cleaning. Hydrophobic cuticular micro grains, characteristic of all Collembola, are useful for coping with the cold, minimizing contact with cold substrates, and counteracting inoculative freezing during severe cold conditions [34].

In the current study, we found that the collected springtails possessed secondary granules, typical of the cuticle structure of the order Poduromorpha. These secondary granules, exhibiting a basic hexagonal structure, are seen as an adaptation to living in soil [33,34]. The Cryo-SEM image also showed that the cuticle granule curvature of the collected springtails (Figure 4C,D) was similar to that of organisms acclimated to winter [35]. Another significant Collembola sensory organ is the post-antennal organ, which has a slightly perforated epicuticle (Figure 4A) and is situated near the eyes (Figure 4B). Usually, these sensory organs are recognized as hygroreceptors [33]. Additional SEM images of the springtails at various magnifications are shown in Figure S3. 

### 3.3. Separation of Protein Based on Molecular Weight 

**Figure 5:**
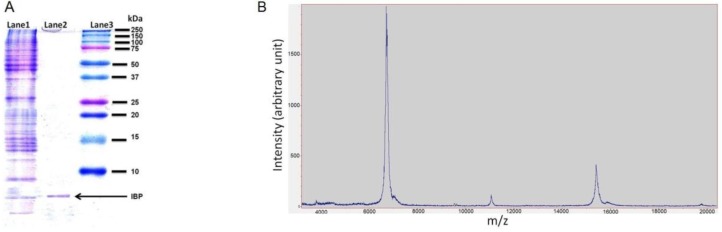
(**A**) Sodium Dodecyl Sulfate-Polyacrylamide Gel Electrophoresis (SDS-PAGE) image of an ice-binding fraction below 10 kDa. Lane 1 shows the crude extra without any purification, lane 2 shows the ice-binding fraction after a two-run of ice-shell purification (IF2), and the lane 3 Precision Plus Protein™ Dual Color Standards (Bio-Rad) (**B**) mass spectroscopy data shows characteristic peaks as 6.5 kDa, 15.7 kDa and an unknown peak at 11.2 kDa.

Based on the centrifugation and size exclusion, the proteins in the snow fleas crude extract were separated. The extract was divided into two fractions: <100 kDa and >100 kDa (as shown in Figure 1). The SDS-PAGE of the small fraction (<100 kDa) indicated the presence of a broad range of proteins (Figure 5; lane 1). The large fraction SDS-PAGE show a protein at the size of 180 kDa, Figure S4. We performed ice-shell purification to isolate the ice-binding molecules present in the small fraction [7]. We repeated the process twice to increase the purification level. A comparison of molecular weights using SDS-PAGE before and after ice-affinity purification indicated the presence of a characteristic 6.5 kDa band (Figure 5; lane 2) after purification, indicative of an AFP, in accordance with the literature [7]. Further, mass spectrometry data also confirms the presence of characteristic 6.5 kDa and 15.7 kDa protein molecules (Figure 5B). Mass spectrometry data also found an unknown peak at 11 kDa.

To further investigate the properties of the two fractions, we performed sets of physical (ice nucleation activity, TH measurements, and ice-shaping activity) and chemical (FTIR and CD spectroscopy) characterizations.

### 3.4. Analysis of Protein Secondary Structure

#### 3.4.1. Fourier-transform infrared spectroscopy (FTIR) Analysis

**Figure 6:**
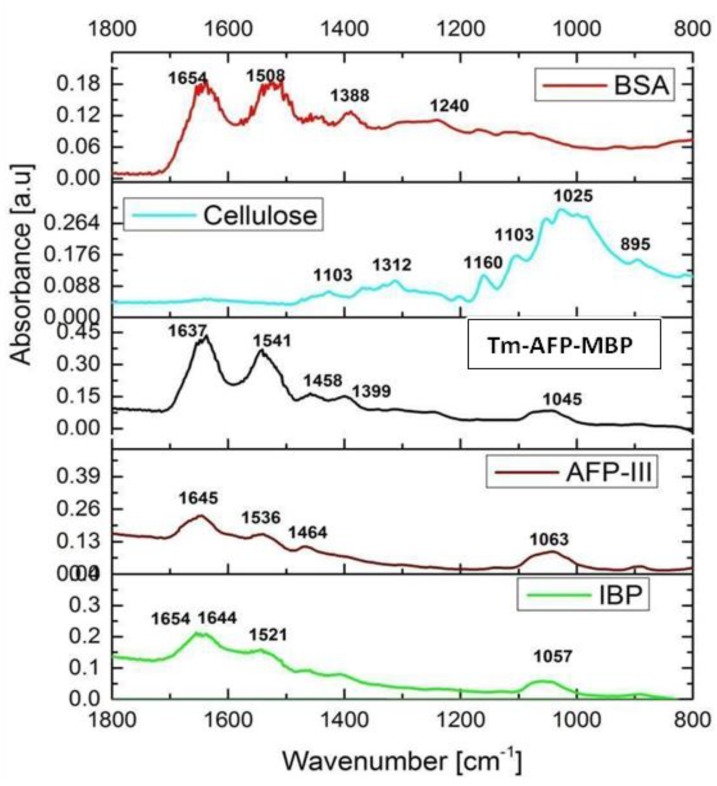
Fourier-transform infrared spectra of the samples between the 1800 and 800 cm^−1^ spectral region. The acronyms used in the figure title: Bovine serum albumin (BSA), Tenebrio molitor antifreeze protein-maltose binding protein chimera (*Tm*AFP*-*MBP), type III fish antifreeze protein (QAE-AFP-III), and the current article purified ice-binding protein (IBP).

The ice-affinity purified fraction was further investigated using FTIR spectroscopy in total reflection mode [36]. The sample was compared with a number of reference compounds, as shown in Figure 6. The ice-affinity purified fractions exhibited strong bands between 1600 and 1700 cm^−1^ and 1520–1590 cm^−1^, which are characteristic bands for the amide I and amide II vibrations, respectively [37]. The samples did not exhibit any pronounced bands between 900 and 1200 cm^−1^ that are characteristic of saccharide moieties, such as cellulose [22], which implied that the molecules were of a proteinaceous nature. Previous studies showed that proteins exhibited absorption bands at 1654 and 1644 cm^−1^, which indicated the presence of an alpha helix and an unordered structure, respectively [38]. The IBP fraction exhibited absorption peaks at 1654, 1644, and 1521 cm^−1^. The peak at 1654 cm^−1^ confirmed the presence of an alpha helix. The existence of the amide I (1645 cm^−1^; positive) and amide II (1543.2 cm^−1^; adverse) bands at wavenumber intervals clearly showed that there was a significant amount of secondary structure-oriented components in the IBP. An absorption band between 1030 and 1040 cm^−1^ was observed in all the samples due to traces of alcohol remaining after the cleaning of the sapphire slide. The FTIR results for the large fraction are shown in Figure S5.

#### 3.4.2. Circular dichroism (CD) Spectroscopy Analysis

**Figure 7:**
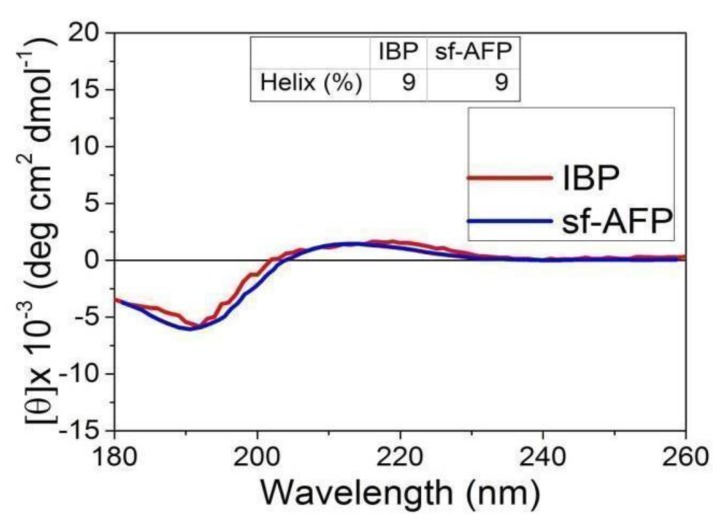
Circular dichroism spectroscopy data of the ice-binding protein (IBP) fraction obtained in the current study and the snow flea 6.5 kDa antifreeze protein reported by [7].

The secondary structure of the protein fractions was determined using CD spectroscopy at 4 °C. In-depth secondary structure analysis was carried out using the Dichro Web Server to plot the CD spectra. The IBP fraction exhibited a typical spectrum, with minima at 195 nm and maxima at 210 nm (see Figure 7). A similar IBP spectrum was obtained from a previously reported snow-fleas antifreeze protein (sfAFP) [7]. The large fraction CD spectroscopy is shown in Figure S6.

### 3.5. Measurement of Thermal Hysteresis (TH)

**Figure 8:**
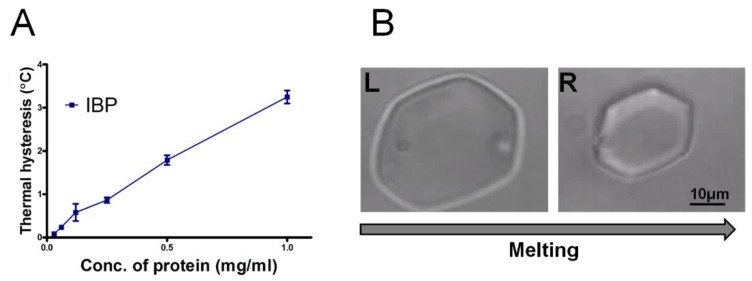
(**A**) Thermal hysteresis activity as a function of the concentration of the ice-binding protein (IBP) fraction after ice-affinity purification. (**B**) Ice morphology in the presence of IBP fraction during melting at a concentration of 0.25 mg/mL (40 µM). The time difference between the L and R image is 1.9 s.

As shown in Figure 8, the TH activity of the IBP fraction is shown as a function of the concentration of protein. The IBP exhibited a TH activity of 1.8 °C at 0.5 mg/mL (80 µM) (Figure 8A), which is similar to the previously reported value of 2.0 °C at 0.3 mg/mL [6]. The ice-crystals retained their shape (Figure 8B, right) within the TH gap until the bursting of the crystal at lower temperature. The TH values indicated that the IBP is a member of the hyperactive AFPs [6,7]. We note that the ice shaped to hexagonal morphology during melting (Figure 8B), which is also a characteristic of hyperactive AFPs in general and of the snow flea AFP in particular (see Figure 4f in [39]). The crystals in Figure 8B have the c-axis perpendicular to the plane of the image. The thermal hysteresis results of the large fraction (>100 kDa) were low, up to 0.1 °C, and are shown Figure S7.

### 3.6. Ice Nucleation Activity

The ability to initiate ice in supercooled nanoliter droplets was determined using the WISDOM setup for IBP solutions of various concentrations in a Tris buffer. The median freezing temperature, T_50_, where 50% of the droplets of pure 50 mM of Tris-HCl and 150 mM of NaCl pH 7 buffer froze and was determined to be −38.2 ± 0.4 °C, which, due to the colligative effect of the dissolved salts, was colder than the T_50_ (−35.7 ± 0.4 °C) of pure water droplets.

**Figure 9:**
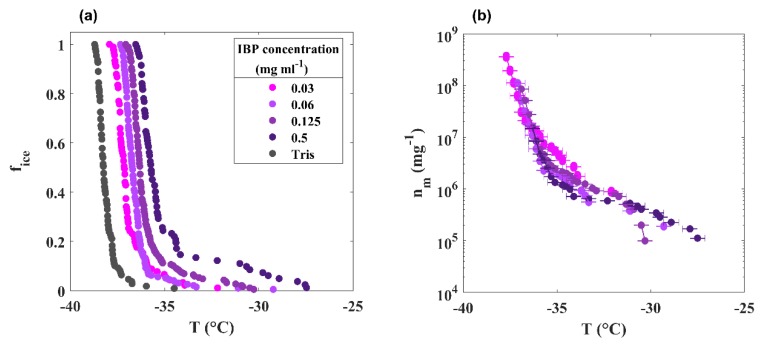
Ice nucleation of IBP solutions. (**A**) Frozen fraction as a function of temperature for various IBP concentrations (purple shades) and the Tris buffer (dark grey). (**B**) The density of ice-active sites (n_m_) per IBP mass as a function of temperature.

Figure 9A shows the cumulative fraction of frozen droplets, f_ice_, as a function of the temperature of the IBP fraction. Four concentrations of IBP were investigated, ranging from 0.03 to 0.5 mg mL^−1^, and a clear dependence of the freezing temperature on the IBP concentration was observed. Higher concentrations exhibited higher freezing temperatures with T_50_ values ranging from −37.2 to −35.7 °C. In Figure 9B, we show the density of ice-active sites (n_m_) per IBP mass as a function of temperature. Higher n_m_ values reflect a more active ice-nucleating material. The n_m_ is based on the cumulative f_ice_ function and was calculated considering the mass of IBP per droplet to account for the effect of IBP concentration on the freezing activity [40,41]. As a result, the data for the four different IBP concentrations, shown as f_ice_ in Figure 8A, all collapsed onto a single n_m_ curve (Figure 9B), clearly supporting the interpretation that the IBPs are indeed the entities that are responsible for the observed ice nucleation. Nucleation experiments of the large fraction (>100 kDa) are shown in Figure S8. 

**Figure 10:**
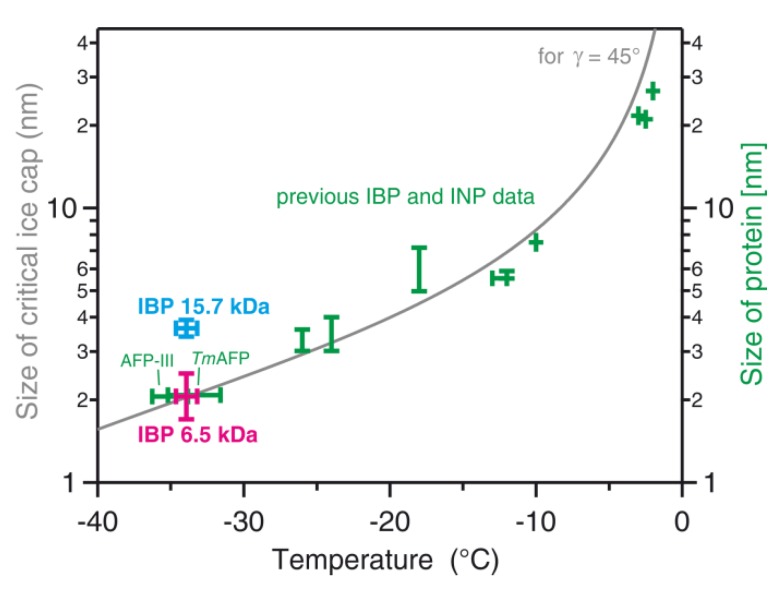
Heterogeneous ice nucleation temperatures for various biological molecular ice nucleators in relation to their size. Magenta = range of median freezing temperatures (adjusted for the effect of the buffer) for Collembolan ice-binding protein (IBP at 6.5 kDa) solutions of different concentrations as determined in the current study. Green = data for IBPs and ice-nucleating proteins (INPs) from previous publications; two recently investigated IBPs from a fish (AFP-III) and an insect (*Tm*AFP) are indicated (see [13] for the data sources). The cyan data point indicates the size of the 15.7 kDa IBP protein at the observed ice nucleation temperature (note that we consider it unlikely that the freezing was indeed due to this 15.7 kDa IBP, see the end of the discussion section in the main text). The grey line corresponds to the size (diameter) of the critical spherical ice cap formed on the protein surface with a contact angle γ of 45° of ice to the protein surface as predicted from CNT calculations (see the text above and Section 3.4 for details).

As presented in Figure 10, the ice nucleation temperatures of the Collembola IBP (magenta) were compared with those of other AFPs and typically much larger ice-nucleating proteins and polysaccharides (green). The ice nucleation temperature range of −34.7 to −33.2 °C of the Collembola IBP is very similar to those of the type-III antifreeze protein from Arctic ocean pout fish (AFP-III in Figure 10) and the hyperactive antifreeze protein from the insect *Tenebrio Molitor* (*Tm*AFP); both were recently measured also using WISDOM [13]. Both of these investigated proteins have similar molecular weights (8.1 kDa and 8.4 kDa for type-III AFP and *Tm*AFP, respectively) to the main IBP extracted from the Collembola and also observed on the SDS-PAGE gel (6.5 kDa), which suggests that their active sites for ice nucleation are of a similar size, estimated to be about 2 nm. In detail, we estimated the size of the ice-binding site of the 6.5 kDa Collembola IBP using the structure given by [6,30]. The rectangular ice-binding site has a size of about 1.7 by 4.7 nm. Such a surface may accommodate a spherical ice cap of 1.7 nm diameter, limited by the shorter length of the ice-binding site. Any larger ice cap must assume an ellipsoidal shape with the two diameters representing the smaller and larger length of the rectangular ice-binding site, i.e., d_1_ = 1.7 nm and d_2_ = 4.7 nm. In order to represent these two ellipsoidal diameters by a single equivalent spherical diameter d_eq_ that can be compared to the spherical ice cap calculation from CNT, we use a version of the Young–Laplace equation, $d_{eq}=2\cdot {\left(1/d_{1}+1/d_{2}\right)}^{-1}$, resulting in a value of d_eq_ = 2.5 nm. Hence, we estimate the size of the 6.5 kDa IBP binding site to be between 1.7 and 2.5 nm, and we have indicated that range in Figure 10 by the vertical magenta bar. Figure 10 also shows, as a grey line, the size (diameter) of the critical spherical ice cap formed on the protein surface with a contact angle γ of 45°, as predicted by CNT calculations [13,40]. The newly obtained ice nucleation data for the Collembola IBP is in accordance with the predicted gray line in Figure 10, similar to the experimental data for the previously investigated AFPs. They are also in the range of ice nucleation temperatures for AFPs of a similar size obtained from recent molecular dynamics simulations using the monoatomic water (mW) model [42].

## 4. Discussion

Collembola species are ubiquitous and their location may play a significant role in their structure and genetics [43]. In this study, a new Collembola species was collected from the Mount Hermon region. According to previous studies [1,33,35], differences in cuticle patterns characterize the Collembola species. Cyro-SEM examination of the Collembola cuticles collected in the current study suggests that they are typical of the order Poduromorpha. Our study reveals that the collected Collembola species also demonstrate a distinctive hydrophobic cuticular micro-grain surface (shown in Figure 4), similar to the previously observed [44]. This cuticular structure may be useful in coping with a cold environment and minimize contact with cold surroundings in Mount Hermon during the winter season.

In order to confirm the morphological characteristics from a taxonomic aspect, phylogenetic analysis was carried out using the COI gene. The COI gene is the most targeted mitochondrial DNA gene due to its high intraspecific diversity, which give it a well-established molecular identification [45]. The morphology and genetic data suggest homogeneity with hypogastruridae species from the order Poduromorpha. In addition, the BOLD analysis also revealed that the species is very closely related to the hypogastruridae species of order Poduromorpha previously reported in Russia [46].

Various studies of Collembola (and other hexapods) that live in cold climates have indicated the presence of ice-nucleating agents and hyperactive AFPs [9]. Here, an antifreeze fraction was separated using size exclusion and ice-affinity purification, resulting in a single band in SDS-PAGE gel. While we expected to see two bands, according to [7], a strong band at 6.5 kDa and a weak band at 15.7 kDa, we observed only the strong band at ~6.5 kDa in SDS-PAGE. Nevertheless, the mass spectrometry measurement shows a strong peak at 6.58 kDa and a weaker peak (20% of the 6.58 peak) at 15.73 kDa. We also observed an unknown weak peak (6% of the 6.58 peak) at 11.22 kDa. This peak, which is close to the average of the two protein masses (6.58 + 15.73)/2 = 11.16, may be an artefact from the mass spectrometer reading. We therefore assume that the larger protein was not observed on the SDS-PAGE gel due to its smaller amount. 

FTIR spectroscopy analysis [35,36] was used to identify the secondary protein structures of the collected protein fractions. The FTIR analysis was performed in the 800 to 1800 cm^−1^ region. The ice shell purified fraction showed spectra at 1600–1700 cm^−1^ and 1520–1590 cm^−1^, which indicated that the ice-binding portion exhibits bands specific to an alpha helix. This result is in accordance with the previously reported ice-binding protein extracted from snow fleas [7]. We further analyzed the protein by a CD band in the far-ultraviolet region, 195–260 nm, which contained the information of the skeleton structure of the proteins. The results indicate that the ice binding protein has a CD spectrum very similar to that of the sfAFP (6.5 kDa), as previously reported [7,47]. We also analyzed the large fraction (>100 kDa). Due to the partial information we obtained for the identification of the proteins, we presented the results of the large fraction in the Supplementary Data.

We examined the TH activity, an important characteristic of AFPs, of our samples. The experiments reveal that the IBPs have a high TH activity of more than 3 °C at a concentration of 1 mg/mL, which confirms that the ice-shell purified proteins are hyperactive AFPs. Our study shows that the crystal shape obtained in the presence of IBPs during melting is consistent with our previous studies on hyperactive antifreeze proteins [39].

In this study, we have shown for the first time the ice nucleation activity of snow flea IBPs. The INA increased with the concentration of IBP in the solution, which led to warmer nucleation temperatures and a higher number of nucleation events at a particular temperature. This study supports previous findings of the contrasting behaviors of AFPs that can inhibit the growth of existing ice crystals and can also initiate the nucleation of new ice crystals in supercooled solutions [13,42,48]. As noted above, we observed two different IBPs in the ice-nucleating fraction: A 6.5 kDa IBP confirmed by SDS-PAGE and mass spectroscopy and a 15.7 kDa IBP confirmed by mass spectrometry. Above, we have attributed the ice nucleation activity to the more abundant 6.5 kDa IBP. Here, we discuss the likelihood that the ice nucleation was actually due to the 15.7 kDa IBP. We did the same analysis described above to determine the representative size of the ice-binding site of the 15.7 kDa IBP. According to Mok et. al. [29], the ice-binding site may be represented by a rectangular site of 3.4 by 4.6 nm. Such a rectangle can accommodate a spherical ice cap of 3.4 nm in diameter or an ellipsoidal ice cap with diameters d_1_ = 3.4 nm and d_2_ = 4.6 nm, resulting in an equivalent spherical diameter of d_eq_ = 3.9 nm. We have added this size range as the cyan vertical range in Figure 10 together with a horizontal temperature range observed in the ice nucleation temperature experiment. Clearly, this 15.7 kDa data point (cyan) is significantly higher than that of the 6.5 kDa IBP (magenta) and, more importantly, is also significantly higher than the grey line obtained from the theory. This comparison implies that ice nucleation by the 6.5 kDa IBP is consistent with calculations of the ice cap radius by CNT, while ice nucleation by the 15.7 kDa IBP would contradict the CNT approach. Hence, we consider it more likely that the 6.5 kDa IBP is indeed the ice-nucleating protein, which is also in agreement with the fact that it is more abundant in the investigated solutions: It gives a strong signal in the SDS-PAGE (see Figure 5A), while the 15.7 kDa IBP does not. In addition, the 6.5 kDa peak in the mass spectrum is substantially more prominent than that of the 15.7 kDa IBP. Nevertheless, we note that a definite proof of this suggestion requires further experiments that we leave for future detailed analysis.

## 5. Conclusions

A new Collembola species was found in Mount Hermon, a snow flea which contains ice-binding proteins (IBPs). The SDS-PAGE and mass spectrometry revealed the presence of a 6.5 kDa IBP, and mass spectrometry also showed small amounts of a 15.7 kDa IBP. These protein masses, as well as the CD spectrum, are similar to those of IBPs extracted from snow fleas collected in Canada [7]. This finding indicates possible conserved snow flea proteins worldwide. Thermal hysteresis measurements indicate that the IBPs are hyperactive antifreeze proteins. In addition, a sensitive nucleation experiment showed a low ice nucleation activity. This ice nucleation activity is most likely due to the smaller but more abundant 6.5 kDa IBP. Therefore, our results support the concept that ice-binding proteins also have heterogeneous ice nucleation activity. As the presence of the larger IBPs in small concentrations seems not to influence the ice nucleation temperature, it may be useful in future studies to investigate the lower detection limit of the ice nucleation activity as a function of concentration.

## Supplementary Materials

**The following are available online at https://www.mdpi.com/2218-273X/9/10/532/s1, **Movie S1: A movie of Snow fleas in the collection jar at the collection site, The COI sequence: published in https://www.ncbi.nlm.nih.gov/nuccore/MN138431, Figure S1: Images of the sampled Collembola in a glass collection tube at the collection site, Figure S2: Agarose gels of PCR products from snow fleas collected from Mount Hermon, Figure S3: SEM image of a springtail in a "normal" fully hydrated state, Figure S4: SDS-PAGE >100kDa fraction, Figure S5: Fourier-transform infrared (FTIR) spectroscopy data for the >100 kDa protein fraction, Figure S6: Circular dichroism spectroscopy data for the >100 kDa protein fraction, Figure S7: Thermal hysteresis activity as a function of the concentration of the >100kDa protein, Figure S8: Ice nucleation of the >100 kDa protein fraction.

## References

[1] Deharveng L., D’Haese C.A., Bedos A., Balian E.V., Lévêque C., Segers H., Martens K. (2008). Global diversity of springtails (Collembola; Hexapoda) in freshwater. Freshwater Animal Diversity Assessment.

[2] Gruia M., Poliakov D., Broza M. (2000). Collembola of Northern Israel, II (Special Papers in Honor of late Professor Ryozo Yoshii). Contrib. Biol. Lab. Kyoto Univ..

[3] Palissa A. (2006). Beiträge zur Collembolenfauna Israels. Beiträge Entomol. Contrib. Entomol..

[4] Montiel P.O., Grubor-Lajsic G., Worland M.R. (1998). Partial desiccation induced by sub-zero temperatures as a component of the survival strategy of the Arctic collembolan Onychiurus arcticus (Tullberg). J. Insect Physiol..

[5] Clark M.S., Thorne M.A., Purać J., Burns G., Hillyard G., Popović Z.D., Grubor-Lajsić G., Worland M.R. (2009). Surviving the cold: Molecular analyses of insect cryoprotective dehydration in the Arctic springtail Megaphorura arctica (Tullberg). BMC Genom..

[6] Lin F.-H., Graham L.A., Campbell R.L., Davies P.L. (2007). Structural modeling of snow flea antifreeze protein. Biophys. J..

[7] Graham L.A., Davies P.L. (2005). Glycine-rich antifreeze proteins from snow fleas. Science.

[8] Braslavsky I., Drori R. (2013). LabVIEW-operated novel nanoliter osmometer for ice binding protein investigations. J. Vis. Exp..

[9] Duman J.G. (2001). Antifreeze and ice nucleator proteins in terrestrial arthropods. Annu. Rev. Physiol..

[10] Worland M.R., Block W. (2003). Desiccation stress at sub-zero temperatures in polar terrestrial arthropods. J. Insect Physiol..

[11] Elnitsky M.A., Hayward S.A.L., Rinehart J.P., Denlinger D.L., Lee R.E. (2008). Cryoprotective dehydration and the resistance to inoculative freezing in the Antarctic midge, Belgica antarctica. J. Exp. Biol..

[12] Block W., Worland M.R. (2001). Experimental studies of ice nucleation in an Antarctic springtail (Collembola, Isotomidae). Cryobiology.

[13] Eickhoff L., Dreischmeier K., Zipori A., Sirotinskaya V., Adar C., Reicher N., Braslavsky I., Rudich Y., Koop T. (2019). Contrasting behavior of antifreeze proteins: Ice growth inhibitors and ice nucleation promoters. J. Phys. Chem. Lett..

[14] Hosoishi S., Ogata K. (2014). Description and DNA barcoding of Crematogaster fraxatrix Forel, 1911 and two new closely related species from Cambodia and Indonesia (Hymenoptera, Formicidae). Zookeys.

[15] Benson D.A., Cavanaugh M., Clark K., Karsch-Mizrachi I., Ostell J., Pruitt K.D., Sayers E.W. (2018). GenBank. Nucleic Acids Res..

[16] Ratnasingham S., Hebert P.D.N. (2007). bold: The Barcode of Life Data System (http://www.barcodinglife.org). Mol. Ecol. Notes.

[17] FigTree. http://tree.bio.ed.ac.uk/software/figtree/.

[18] Biggs C.I., Bailey T.L., Graham B., Stubbs C., Fayter A., Gibson M.I. (2017). Polymer mimics of biomacromolecular antifreezes. Nat. Commun..

[19] Marshall C.J., Basu K., Davies P.L. (2016). Ice-shell purification of ice-binding proteins. Cryobiology.

[20] Adar C., Sirotinskaya V., Bar Dolev M., Friehmann T., Braslavsky I. (2018). Falling water ice affinity purification of ice-binding proteins. Sci. Rep..

[21] Sullivan S.T., Tang C., Kennedy A., Talwar S., Khan S.A. (2014). Electrospinning and heat treatment of whey protein nanofibers. Food Hydrocoll..

[22] Dreischmeier K., Budke C., Wiehemeier L., Kottke T., Koop T. (2017). Boreal pollen contain ice-nucleating as well as ice-binding “antifreeze” polysaccharides. Sci. Rep..

[23] Johnson W.C. (1999). Analyzing protein circular dichroism spectra for accurate secondary structures. Proteins.

[24] Whitmore L., Wallace B.A. (2008). Protein secondary structure analyses from circular dichroism spectroscopy: Methods and reference databases. Biopolymers.

[25] Reicher N., Segev L., Rudich Y. (2018). The WeIzmann Supercooled Droplets Observation on a Microarray (WISDOM) and application for ambient dust. Atmos. Meas. Tech..

[26] Reicher N., Budke C., Eickhoff L., Raveh-Rubin S., Kaplan-Ashiri I., Koop T., Rudich Y. (2019). Size-dependent ice nucleation by airborne particles during dust events in the eastern Mediterranean. Atmos. Chem. Phys..

[27] Koop T., Zobrist B. (2009). Parameterizations for ice nucleation in biological and atmospheric systems. Phys. Chem. Chem. Phys..

[28] Attard E., Yang H., Delort A.M., Amato P., Pöschl U., Glaux C., Koop T., Morris C.E. (2012). Effects of atmospheric conditions on ice nucleation activity of *Pseudomonas*. Atmos. Chem. Phys..

[29] Mok Y.-F., Lin F.-H., Graham L.A., Celik Y., Braslavsky I., Davies P.L. (2010). Structural basis for the superior activity of the large isoform of snow flea antifreeze protein. Biochemistry.

[30] Pentelute B.L., Gates Z.P., Tereshko V., Dashnau J.L., Vanderkooi J.M., Kossiakoff A.A., Kent S.B.H. (2008). X-ray structure of snow flea antifreeze protein determined by racemic crystallization of synthetic protein enantiomers. J. Am. Chem. Soc..

[31] Fanciulli P.P., Summa D., Dallai R., Frati F. (2001). High levels of genetic variability and population differentiation in *Gressittacantha terranova* (Collembola, Hexapoda) from Victoria Land, Antarctica. Antarct. Sci..

[32] Hogg I.D., Hebert P.D. (2004). Biological identification of springtails (Hexapoda: Collembola) from the Canadian Arctic, using mitochondrial DNA barcodes. Can. J. Zool..

[33] Helbig R., Nickerl J., Neinhuis C., Werner C. (2011). Smart skin patterns protect springtails. PLoS ONE.

[34] Hensel R., Neinhuis C., Werner C. (2016). The springtail cuticle as a blueprint for omniphobic surfaces. Chem. Soc. Rev..

[35] Gundersen H., Thaulow C., Leinaas H.P. (2015). Seasonal change in the wetting characteristics of the cuticle of the Collembola Cryptopygus clavatus (Schött, 1893). Zoomorphology.

[36] Gonzalez L., Wade M., Bell N., Thomas K., Wess T. (2013). Using attenuated total reflection Fourier transform infrared spectroscopy (ATR FT-IR) to study the molecular conformation of parchment artifacts in different macroscopic states. Appl. Spectrosc..

[37] Cunha A.V., Bondarenko A.S., Jansen T.L.C. (2016). Assessing spectral simulation protocols for the amide I band of proteins. J. Chem. Theory Comput..

[38] Kennedy D.F., Crisma M., Toniolo C., Chapman D. (1991). Studies of peptides forming 3(10)- and alpha-helices and beta-bend ribbon structures in organic solution and in model biomembranes by Fourier transform infrared spectroscopy. Biochemistry.

[39] Bar-Dolev M., Celik Y., Wettlaufer J.S., Davies P.L., Braslavsky I. (2012). New insights into ice growth and melting modifications by antifreeze proteins. J. R. Soc. Interface.

[40] Budke C., Koop T. (2015). BINARY: An optical freezing array for assessing temperature and time dependence of heterogeneous ice nucleation. Atmos. Meas. Tech..

[41] Zobrist B., Koop T., Luo B.P., Marcolli C., Peter T. (2007). Heterogeneous ice nucleation rate coefficient of water droplets coated by a nonadecanol monolayer. J. Phys. Chem. C.

[42] Qiu Y., Hudait A., Molinero V. (2019). How Size and Aggregation of Ice-Binding Proteins Control Their Ice Nucleation Efficiency. J. Am. Chem. Soc..

[43] Cicconardi F., Fanciulli P.P., Emerson B.C. (2013). Collembola, the biological species concept and the underestimation of global species richness. Mol. Ecol..

[44] Eisenbeis G., Meyer E., Margesin R., Schinner F. (1999). Ecophysiological and morphological features of glacier-dwelling Collembola. Cold-Adapted Organisms.

[45] Hebert P.D.N., Ratnasingham S., deWaard J.R. (2003). Barcoding animal life: Cytochrome c oxidase subunit 1 divergences among closely related species. Proc. Biol. Sci..

[46] Rougerie R., Decaëns T., Deharveng L., Porco D., James S.W., Chang C.-H., Richard B., Potapov M., Suhardjono Y., Hebert P.D.N. (2009). DNA barcodes for soil animal taxonomy. Pesq. Agropec. Bras..

[47] Pentelute B.L., Gates Z.P., Dashnau J.L., Vanderkooi J.M., Kent S.B.H. (2008). Mirror image forms of snow flea antifreeze protein prepared by total chemical synthesis have identical antifreeze activities. J. Am. Chem. Soc..

[48] Mochizuki K., Qiu Y., Molinero V. (2017). Promotion of homogeneous ice nucleation by soluble molecules. J. Am. Chem. Soc..

